# Supplementary material for: N6-Methyladenosine RNA Modification Regulates the Differential Muscle Development in Large White and Ningxiang Pigs
Source: Cells. 2024 Oct 21;13(20):1744. doi: 10.3390/cells13201744 (PMC11506082; doi:10.3390/cells13201744)
Supplement: Supplementary file 1 [file cells-13-01744-s001.zip › supporting information_╕▒▒╛.pdf]

Table S1. Primer sequences required in experiments.

Gene	Sequence (5′ to 3′)
ALKBH5	F: TTCAAGCCTATCCGGGTGTC<br>R: TCAGCAGCATATCCACTGAG
BEST3	F: TCTTCTTCGCGTGCCTGATT<br>R: TAAGCTGCTCGGCTACCTTG
DNAJBI	F: AGGACCATACCCGTTGTGTT<br>R: AGGACGGTTCTTGAGGTCTG
EIDI	F: ATGGTTGTCAATCGTCTG<br>R: GGAATGTTGTTGGGTCTC
EIF2AK3	F: GCAGTGGCAACGAGAAGTGGAA<br>R: CGTGGCAGCTTCCTGTTCTTCC
FTO	F: CCCTGCAGAATGTCCGTGAT<br>R: CCACTCAAACTCGACCTCGT
HSPA6	F: GAATCCGCAGAATACCGTGT<br>R: TCCGCAGTCTCCTTCATCTT
HSPHI	F: CTGAACCTCCTCACCAGAATG<br>R: GTCTCAGTATGTTATGTAGAATCAC
LOC100157	F: ACCAGCGAAGAACAGACTGG<br>R: CTGTCGTCGCCCACTTAGAG
LOC106509550	F: ACACCCATAGCCCTAATC<br>R: CACGACCTTGACACTTTT
LOC110261121	F: ACAAAGGAGAGGAAAGGCCC<br>R: TGGACCTGCGTTTGTGTGAA
LOC100518848	F: CAATAACTGACTTTCTGCGCTC<br>R: GGCACGTATAGGTCCACGAA
LOC110259172	F: CCCATCGACATCCTCTTCG<br>R: GGACTGCCCAGGCTCAAAC
LOC100158003	F: CGACCTGATGTATGCCAAGA<br>R: GCCCACCTCCTCATAATCC
LOC110257607	F: AGATGCCACAGCCTTGAGTC<br>R: ACCTAGCATAGCTGAACGGC
MAP7D2	F: TCAGCCATGAATTTACCAA<br>R: TCAACAGTCGCATAACAAGA
METTL3	F: ATCGTAGCCGAGGTTCGTTC<br>R: AATCTTTCGAGTGCCAGGGG
METTL14	F: AGATTGCAGCTCCTCGATCA<br>R: CCCACTGCGTAAACACACTC
RAB3C	F: TGATGTAGAAGGTGCGTGAA<br>R: GCTGTTGGTGGGAAAGTG
SH3BP4	F: CCAAGGTGTCCAAGCAGCAGAT<br>R: TGGTGGCTCCAGGTGAGTAGAA

TULP2	F: TGCCTTGCCACCTCCTTA<br>R: GCACCTCGTATCCGTCTC
WFS1	F: GCCAGGAGCCAAGAAAGA<br>R: AGTCCACGGCAGTGCAAT
WTAP	F: GGCCAACGGACCAAGTAATG<br>R: TCATGTGAGTGGCGTGTGA
YTHDF2	F: ATGCCTCGGCCATTGTGTG<br>R: CGCCGAGAGAAGGGAACAC

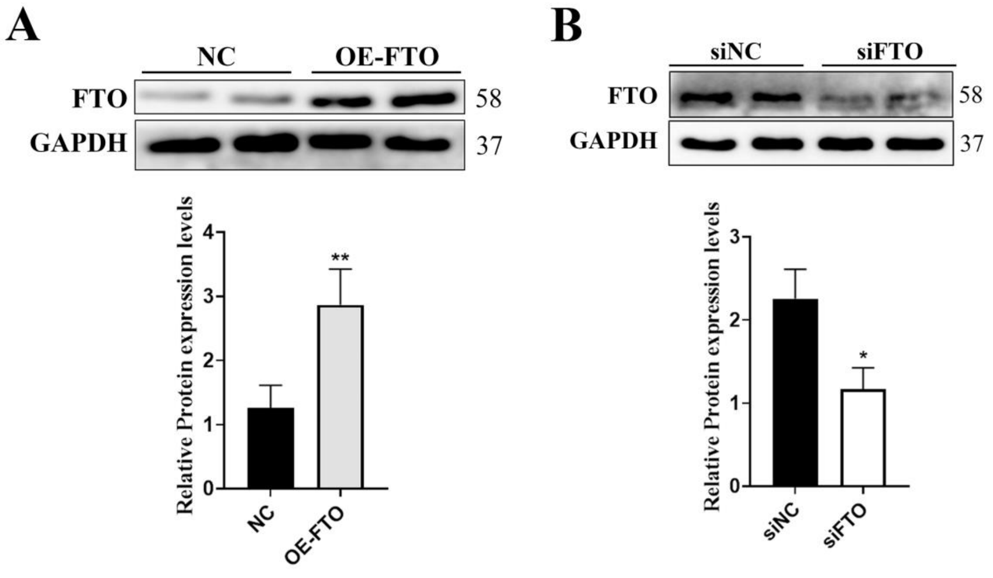

Figure S1. Efficiency of overexpression and interference with FTO. (A) Protein expression and densitometric analysis of FTO in PSCs after overexpression of FTO. (B) Protein expression and densitometric analysis of FTO after siRNA interference.

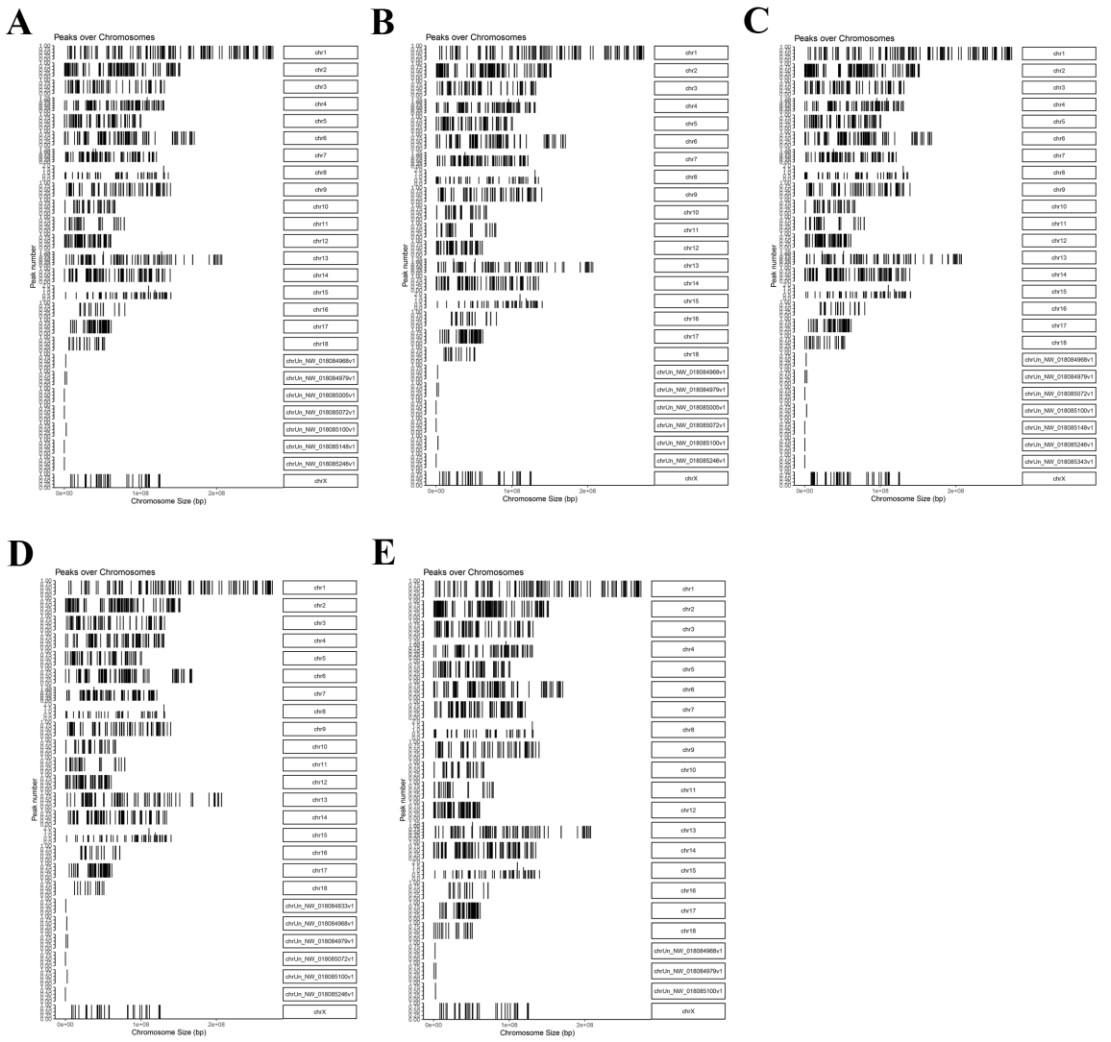

Figure S2. The distribution of m^{6}A enriched regions on the chromosomes of each pig. (A) LW-1, (B) LW-2, (C) LW-3, (D)NX-2, (D)NX-3.

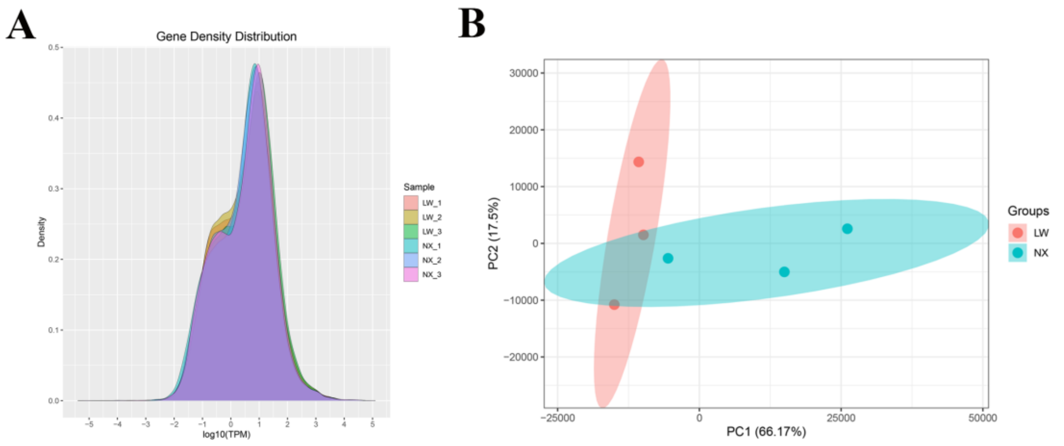

Figure S3. Densitometric and cluster analysis of gene expression profiles. (A) Gene density distribution of all samples. (B) Cluster analysis of LW group and NX group.

SacI SalI

MutC: GAGCTCAGCGAGTTCAAGGACGTGCTGCTGCACCTGGTCGAC

MutT: GAGCTCAGCGAGTTCAAGGATGTGCTGCTGCACCTGGTCGAC

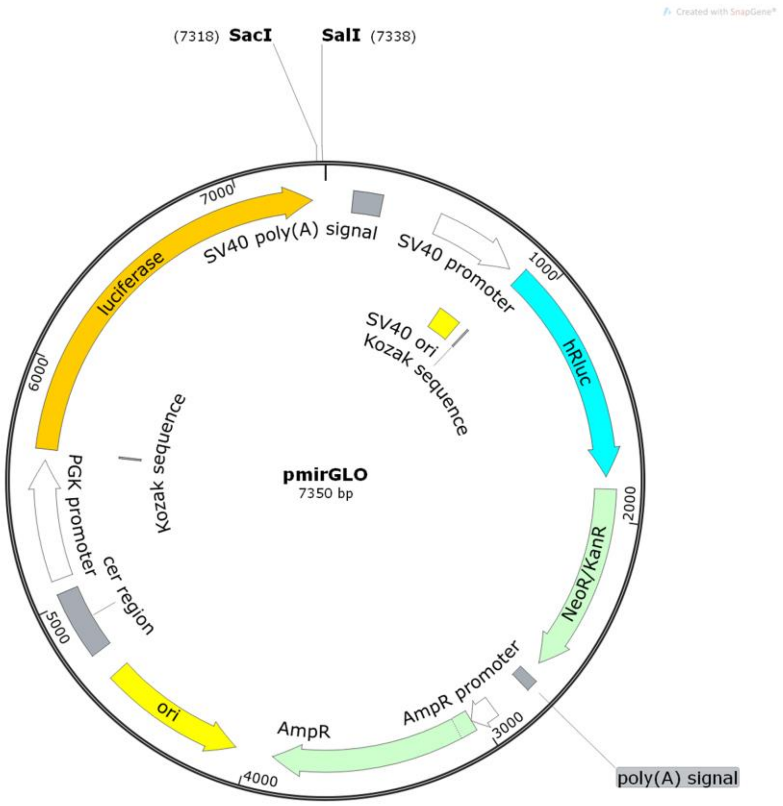

Figure S4. PrmiGLO-MUTC and prmiGLO-MUTT vector sequences.
